# Splenic Perforation Following Colonoscopy

**DOI:** 10.4021/gr2009.05.1292

**Published:** 2009-05-20

**Authors:** Lakshmi Pasumarthy, James Srour

**Affiliations:** a Department of Medicine, York Hospital, 1001 S. George St, York, PA 17405, USA; b Department of Gastroenterology, York Hospital, 1001 S. George St, York, PA 17405, USA

**Keywords:** Splenic Perforation, Colonoscopy

## Abstract

Splenic perforation represents a rare complication of colonoscopy. In our report we have reviewed the experience reported in the world literature, including proposed mechanisms, risk factors for splenic perforation and available management options. We have also discussed our concerns for under reporting. We had a total of 4 cases of splenic perforation following colonoscopies at our centre. One patient had a small splenic laceration repaired; two were observed closely and discharged without intervention. The patient mentioned below required a splenectomy.

## Introduction

Colonoscopy is widely accepted as a common investigation for the diagnosis and treatment of various colorectal conditions, in addition to screening and surveillance of colorectal neoplasms. It is generally safe. The most common complications include colonic perforation and hemorrhage. Perforation of other organs is extremely rare.

## Case report

A 75 year old male underwent colonoscopy for the indication of increasing constipation. Anesthesia was induced with 160 mg of propofol, 50 mcg of fentanyl and 20 mg of Ketamine. He was found to have four dimunitive polyps which were removed by hot snare, two in the hepatic flexure, one in the distal colon, one in the proximal right colon. He also had extensive diverticular disease without inflammation. He tolerated the procedure and was discharged home. Early next morning he had generalized pain abdomen which became steadily worse.

His past medical history was significant for right upper lobe lung cancer, for which he had a right upper lobe ablation and subsequently chemotherapy and radiation therapy, hypertension, chronic obstructive pulmonary disease with 50 pack years of smoking. He had partial gastrectomy and vagotomy performed twenty years ago peptic ulcer disease. His medications included pantoprazole, amlodipine, albuterol/atrovent, fluticasone/ salmeterol, aspirin. He did not discontinue aspirin prior to the colonoscopy.

When he was brought into the emergency room his vital signs were: BP 62/52 mmHg, pulse rate 93/min, respiratory rate 40/min, temperature 36.4°C, saturation of 100% on 2 liters nasal cannula. He was in the Trendelenburg position, in pain, pale and sweaty. Heart sounds were clear, air entry was reduced to bases. There was no shoulder tenderness (Kehr’s sign). Inspection of abdomen showed an old mid line scar. There was some tenderness palpated in the right lower quadrant and hypogastric region, bowel sounds were well heard. Rectal exam showed guaiac negative stool.

Laboratory work showed white cell count 1.07 X 10^4^/mm^3^, hemoglobin 6.8 g/ dL, hematocrit 20.3 %, platelets 8.03 X 10^5^/mm^3^, lactate 2.3 mml/L. X- ray of abdomen showed non specific bowel gas pattern.

**Figure 1:**
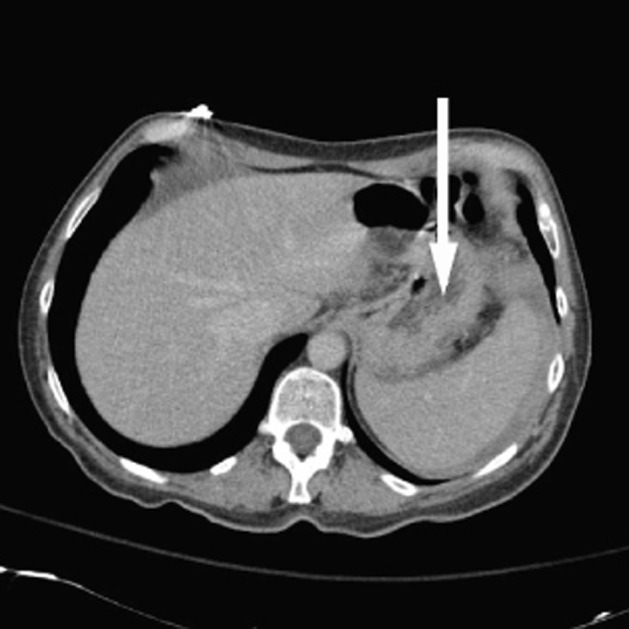
Deformed spleen with active extravasation

CT scan showed evidence of hemoperitoneum with active extravasation from the spleen in addition to left colonic and sigmoid diverticulosis (Fig. 1). The patient was taken to the operating room and underwent urgent laparotomy. A large hematoma was found and evacuated. The spleen was noted to have ruptured and was therefore removed. Adhesions were encountered and lysed. No colonic perforation was noted. The rest of the abdominal exploration was unremarkable and the incision was closed. The patient did receive 3 liters of crystalloid during the procedure and 2 units of packed red blood cells. Histopathology confirmed a ruptured spleen with no evidence of malignancy.

Following the procedure, the patient received splenic vaccines as per the protocol. His hemoglobin and hematocrit were followed daily and they remained stable. He was discharged home in a good condition on the eighth day and continues to do well.

## Discussion

Approximately 1.27 million colonoscopies are performed by gastroenterologists annually for colorectal cancer screening-related indications. When one increases this number by 33% to include colonoscopies not done by gastroenterologists it leads to an estimate of 1.69 million screening-related colonoscopies each year in USA [1].

Colonoscopy is well tolerated, generally safe and widely used for diagnosis and treatment of various colorectal conditions. It is not without complications. The most common ones are colonic perforation (0.34 - 2.14%) and hemorrhage (1.8 - 2.5%). The variation is dependent on the centers reporting and most importantly if polypectomy accompanied the procedure. The first case of splenic perforation following colonoscopy was described by Wherry and Zehner in 1974 [2]. We used Medline for research purpose and identified 54 prior cases, in addition to the ones at our centre. Females sex seems to predominate with a ratio of 3.8:1, the elderly seem to be slightly more susceptible than the young, with a mean age of 62 years. 46% had polypectomy with their colonoscopy, 59% had history of prior abdominal surgery, 55% has diagnosis confirmed by CT scan and 71% required splenectomy [3]. The majority of cases present within a day or two from the injury but delays as much as 13 days have been reported [4].

Patients with splenic rupture commonly present with abdominal pain and symptoms of hypotension, although asymptomatic splenic rupture has been reported [5]. Hemodynamic status directly correlates with extent of rupture and determines the management.

The proposed mechanisms of splenic injury during colonoscopy include excessive traction on the splenocolic ligament which can avulse the splenic capsule, as well as direct trauma to the spleen with the colonoscope [6, 7] as when the endoscope traverses the splenic flexure. Other well recognised mechanisms of injury include splenomegaly, anticoagulation, inflammatory bowel disease, technically difficult colonoscopy, therapeutic colonoscopy, and intra-abdominal adhesions secondary to prior abdominal or pelvic surgery [8, 9]. Conditions that cause splenomegaly such as infections with malaria, typhoid fever, infectious mononucleosis, or leukemic infiltration of the spleen can theoretically pose as additional risk factors.

Some colonoscopic manipulations such as slide by, alpha maneuver [10] and straightening of the sigmoid loop can increase the chances of rupture by increasing the traction on the splenocolic ligament. Technical difficulty encountered during colonoscopy is sometimes decreased by application of external pressure by an assistant. This may cause direct trauma to an enlarged spleen or reduce the relative mobility between spleen and colon.

When the patient assumes the supine position the forces exerted on the spleen due to gravity and traction during colonoscopy oppose each other. This factor will increase the chance of the splenic capsule tearing, especially if there are other predisposing factors, such as previous abdominal surgery. To prevent this complication, patients belonging to the high-risk group should be placed in the left lateral position [11].

Are we using sedation to our disadvantage? In the past, the endoscopist would use symptoms of pain and discomfort as a critical feedback mechanism for determining how well the patient was tolerating the procedure. With the presence of an anesthetist, however, there is a temptation to simply treat these complaints with more medication rather than modify the technique. Before the administration of additional sedation or analgesia, other modifying measures must be attempted, such as desufflation, retracting the colonoscope, changing the patient’s position.

Heightened awareness of the complications of a procedure, its manifestations and predisposing factors are all very important in recognising and managing the problem. Rapid evaluation of the critically ill patient, judicious resuscitation reduces morbidity and prevents mortality.

Kehr sign, referred left shoulder pain due to diaphragmatic irritation, is reported to occur in 90% of cases of splenic rupture [12]. Unfortunately, it is also reportedly present in 50% of patients who have undergone uncomplicated colonoscopies, thereby limiting its usefulness [13].

Anemia (76%) and leucocytosis (74%) are the most common laboratory value abnormalities. Abdominal X- ray is often inconclusive. CT scan is the imaging modality of choice since it determines the extent of splenic damage, is better at picking up hemoperitoneum [14]. This will guide the management of patient as in our case. Ultrasonography can be useful for quickly identifying free fluid in the abdomen in the unstable patient, although gas in the bowels after colonoscopy may limit its usefulness. Only the first reported case was diagnosed after angiography.

Conservative management usually includes inpatient/ICU monitoring, serial hemoglobin checks, and serial examinations. Approximately 70% required splenectomy which is performed when there is evidence of hemodynamic instability, underlying splenic disease; a grade III traumatized spleen by CT, and hemoperotineum. Two cases of colonoscopy induced splenic injury have been successfully treated with angiographic embolization [15, 16]. Overall, 27% of patients who have sustained colonoscopic splenic rupture were treated nonoperatively. Three deaths have occurred [17].

Finally, there is a concern for under-reporting of complications. As described by Zubarik and colleagues [18], more complications were detected by contacting patients 30 days after outpatient colonoscopy than were discussed at patient safety conferences indicating that physician self-report of complications tends to under report complications compared with systematic follow-up. There is still a lack of consensus as to what constitutes a complication and how to define it. Moreover, providers who are now being told that their outcomes data will be made publicly available and will serve as basis for changes in their payments and referral patterns have an incentive to under report the complications. This especially happens when the interpretation of what constitutes a complication is left to the discretion of the individual provider [19]. With introduction of large multicenter databases such as the CORI (Clinical Outcomes Research Initiative) project better estimates of complications should be available in future.
